# The 5'-end transitional CpGs between the CpG islands and retroelements are hypomethylated in association with loss of heterozygosity in gastric cancers

**DOI:** 10.1186/1471-2407-6-180

**Published:** 2006-07-10

**Authors:** Young-Ho Kim, Seung-Jin Hong, Yu-Chae Jung, Sung-Ja Kim, Eun-Joo Seo, Sang-Wook Choi, Mun-Gan Rhyu

**Affiliations:** 1 Department of Microbiology, College of Medicine, The Catholic University of Korea, Seoul, Korea; 2 Department of Clinical Pathology, College of Medicine, The Catholic University of Korea, Seoul, Korea; 3 Department of Internal Medicine, College of Medicine, The Catholic University of Korea, Seoul, Korea

## Abstract

**Background:**

A loss of heterozygosity (LOH) represents a unilateral chromosomal loss that reduces the dose of highly repetitive *Alu*, L1, and LTR retroelements. The aim of this study was to determine if the LOH events can affect the spread of retroelement methylation in the 5'-end transitional area between the CpG islands and their nearest retroelements.

**Methods:**

The 5'-transitional area of all human genes (22,297) was measured according to the nearest retroelements to the transcription start sites. For 50 gastric cancer specimens, the level of LOH events on eight cancer-associated chromosomes was estimated using the microsatellite markers, and the 5'-transitional CpGs of 20 selected genes were examined by methylation analysis using the bisulfite-modified DNA.

**Results:**

The extent of the transitional area was significantly shorter with the nearest *Alu *elements than with the nearest L1 and LTR elements, as well as in the extragenic regions containing a higher density of retroelements than in the intragenic regions. The CpG islands neighbouring a high density of *Alu *elements were consistently hypomethylated in both normal and tumor tissues. The 5'-transitional methylated CpG sites bordered by a low density of *Alu *elements or the L1 and LTR elements were hypomethylated more frequently in the high-level LOH cases than in the low-level LOH cases.

**Conclusion:**

The 5'-transitional methylated CpG sites not completely protected by the *Alu *elements were hypomethylated in association with LOH events in gastric cancers. This suggests that an irreversible unbalanced decrease in the genomic dose reduces the spread of L1 methylation in the 5'-end regions of genes.

## Background

Unilateral chromosomal losses detected by a loss of heterozygosity (LOH) analysis are the most common genetic events in solid tumors [1]. The level of chromosomal losses, rather than a single loss, has been consistently shown to be a significant predictor of survival in stage II and III gastrointestinal cancer patients [2-6]. Although chromosomal losses are a part of gene inactivation, in a naïve sense, the LOH events represent a reduction in the dose of genetic elements. A dosage compensation mechanism, which maintains the dose of the human genome, equalizes the difference in the dose of the X chromosome between males (XY) and females (XX) via pan-chromosomal epigenetic inactivation [7-9]. Therefore, it is important to determine if the level of LOHs underlying the clinical course of gastric cancer is associated with the epigenetic changes occurring in tumor cells as a result of an unbalanced reduction of the genomic dose.

The distributions of the two major repetitive sequences in the human genome, the *Alu *and L1 retroelements, are differentially biased toward the CpG-rich euchromatic and CpG-poor heterochromatic regions, respectively [10]. These self-replicating sequences are suppressed by hypermethylation because the vegetative copy generates integrative copies resulting in the harmful influence of insertion mutagenesis. In addition, the retroelements are known to be potential centres for the spread of CpG methylation to the adjacent CpG sites [11-14]. The L1 elements can boost the long-distance spread of heterochromatin that is responsible for chromosome X inactivation [7,15]. Meanwhile, high-density *Alu *elements near the CpG-rich islands are believed to spread CpG methylation over short distances [16]. The short-distance spread of *Alu *methylation and the long-distance spread of L1 methylation appear to construct the two distinct chromatin structures.

Repetitive sequences in eukaryotes can initiate and facilitate the spread of RNA-mediated chromatin condensation through the pairing of homologous sequences [17]. Given the interdependence of CpG methylation and chromatin condensation, human retroelements can also cause the spread of RNA-mediated methylation [15,18]. An analysis of the relationship between the length of the CpG islands and the position of the nearest retroelement revealed that the spread of retroelement methylation can influence the size of a CpG island [16]. Unilateral chromosomal losses frequently occurring in tumor cells are accompanied by an irreversible unbalanced decrease in the dose of genomic and transcriptional retroelements. The colorectal cancer genome suffering LOH events undergoes global hypomethylation that affects the L1 retroelements [19]. In addition, a study of gastric cancers reported a close relationship between the high-level LOHs and the hypomethylation changes in the methylated CpGs near the CpG islands [20]. Therefore, the LOH events might reduce the spread of retroelement methylation as well as the methylation of the retroelement copies.

In this study of gastric cancers, the relationship between the LOH events and the spread of retroelement methylation was examined by determining the correlation between the extent of LOHs and the transitional CpG methylation between the CpG islands and retroelements in the 5'-end regions. The extent of the transitional area was found to be shorter with the nearest *Alu *elements than with the nearest L1 and LTR elements. The 5'-transitional methylated CpG sites bordered by a low density of *Alu *elements or L1 and LTR elements were frequently hypomethylated in the high-level LOH cases. This suggests that unilateral chromosomal losses reduces the spread of L1 methylation.

## Methods

### Computational analyses of the human genome

Information on the coding and non-coding sequences as well as CpG islands was obtained from the human genome database in the May, 2004 version [21]. A 1-kb sized non-overlapping window was used to analyze the sequence characteristics of a 10-kb segment upstream and downstream from each transcription start site. This window analysis allowed an examination of the major regulatory regions, which are usually rich in protein binding motifs, and at the same time, produced a profile of the 5'-transitional CpGs between the CpG islands and their nearest retroelements. The sequence data delimited from the promoter regions were input into a local program to calculate the coverage of CpG islands. A CpG island was defined as a DNA segment with a G+C content ≥ 50%, longer than 200 bp nucleotides, and an Observation/Expectation CpG ratio > 0.6 [22]. A similar procedure was used for the genomic position and annotation of retroelements using the RepeatMasker program [23].

### Patients and tumor tissues

The gastric cancer tissue and non-cancerous mucosa were obtained from 50 patients who had undergone surgery between March and December 2003 at St. Paul's Hospital, The Catholic University of Korea. These fresh archives provided the genomic DNA that had been amplified efficiently by methylation-specific PCR (MSP). The histological classification of the gastric cancers was carried out according to the Lauren's classification [24,25], and the degree of differentiation was graded according to the recommendations of the World Health Organization for the histological typing of gastric cancer [26]. The Tumor-Node-Metastasis (TNM) criteria were used to determine the tumor stage based on the pathological and clinical findings observed from radiography, ultrasonography, computed tomography, and abdominal exploration during laparotomy. The Institutional Review Board approved this study, and written informed consent was obtained from each patient prior to the surgical resection.

A single 5–7 mm diameter tumor site containing a homogeneous cell content was selected from each representative tissue section. Seven-μm-thick hematoxylin-eosin-stained sections were microdissected under a 40-x stereomicroscope using a surgical scalpel. All the microdissected tumor sites were checked for a tumor cell content ≥ 70% prior to DNA extraction. A major fraction (33 sites, 66%) of the microdissected tissues contained a tumor cell content of 80–89%, followed by ≥ 90% (12 sites, 24%) and 70–79% (5 sites, 10%). Approximately 50 microdissected cells were digested in 1 μl of a Tween 20 – Proteinase K lysis buffer. An average of 100 μl of each microdissected tissue lysate was used for microsatellite analysis, and an average of 500 μl of the same lysate was subjected to bisulfite modification for methylation analysis.

### PCR-based microsatellite analysis

**Figure 1:**
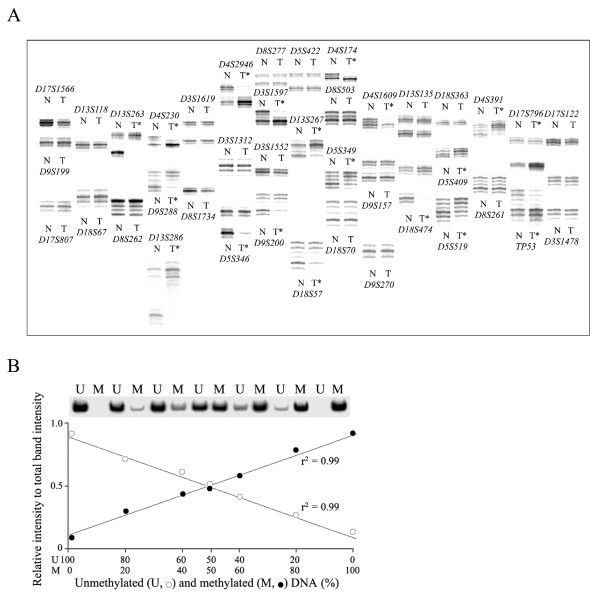
**Representative autoradiographs of microsatellite analysis (A) and standard curves for methylation and unmethylation MSP amplification (B) using a radioisotope**. (A) A "multiplex, hot-start" method was applied to the PCR-based loss of heterozygosity (LOH) analysis. Forty microsatellite allelic loci were amplified in 25 reaction mixtures each of which contained one (10 mixtures) or two (15 mixtures) pairs of primers. A mixture of different-sized two to four amplicons was loaded onto one lane. A total of 80 microsatellite amplicons from each specimen were run simultaneously on one sequencing gel. Case 1 had high-level LOHs involving chromosome 4p, 5q, 9p, 13q, 17p, and 18q. The normal (N) and the corresponding tumor (T) DNAs are indicated above or below each microsatellite amplicon. The asterisk indicates a LOH. (B) The genomic DNA universally methylated by DNA methylase (CpGenome Universal Methylated DNA, Chemicon, Temecula, CA) was used as the methylated control DNA. The PCR DNA, which had been amplified by the universal primer (5'-CCG ACT CGA GNN NNN NAT GTG G-3'), was used as the unmethylated control DNA. Variable mixtures of the two opposite control DNAs according to their PCR intensity of 20 ng/μl were amplified using a set of MSP primers for the non-island CpGs of the *TFF2 *gene. The proportion of methylated and unmethylated CpGs was calculated using the following formula: Methylation or unmethylation proportion (%) = (methylation or unmethylation intensity/(methylation + unmethylation intensity)) × 100. The relative band intensities of the methylation (M, closed circle) and unmethylation (U, open circle) primer set were plotted as a function of the control DNA content. The amplification intensity ratio of the MSP primer set increased linearly with increasing percentage of the corresponding control DNA in the MSP mixtures.

The MSI (microsatellite instability) and LOH status of each gastric cancer was determined using a panel of 40 microsatellite markers on eight cancer-associated chromosomes, 3p, 4p, 5q, 8p, 9p, 13q, 17p, and 18q, as reported elsewhere (figure 1A) [5,6,27]. The MSI status of the microsatellite sequences was scored if there were any frameshift mutations in > 40% of the homozygous markers. The unilateral reduction of the heterozygous allelic bands was interpreted as LOH. The level of LOHs representing a significant reduction in the genomic dose was determined based on the number of chromosomes showing more than one LOH. According to the genetic classification criterion of gastric cancers [6,27], the level of LOHs was divided into low (LOH-L, less than four) and high (LOH-H, four or more losses) levels for the intestinal-type cancers, and baseline (LOH-B, zero or one loss), low (LOH-L, two or three losses), and high (LOH-H, four or more losses) levels for the diffuse-type cancers.

### DNA modification by sodium bisulfite

Because the quality of the formalin-fixed tissue DNA varied, the amount of template DNA was determined based on a PCR band intensity of 20 ng/μl that had been amplified by the microsatellite primer set, *D19S226 *(forward, 5'-CCA GCA GAT TTT GGT GTT GTC TA-3'; reverse, 5'-ACA GAG CCA GAG CCA GTA GGA GT-3'; amplicon size, 164 bp). Ninety microliters of the genomic DNA was denatured with 10 μl of 3 M NaOH for 15 min at 37°C before sodium bisulfite modification. This procedure involved modifying 100 μl of the denatured DNA with 1,040 μl of 2.3 M sodium bisulfite and 60 μl of 10 mM hydroquinone for 12 hr at 50°C, as described elsewhere [20]. The modified DNA was then purified using a genomic DNA purification kit (Promega, Madison, WI, USA), precipitated with ethanol, and dissolved in 35 μl of 5 mM Tris buffer (pH 8.0). One μl aliquot of the modified DNA solution was placed in the PCR tube and stored at -20°C.

### Semiquantitative methylation analysis

**Figure 2:**
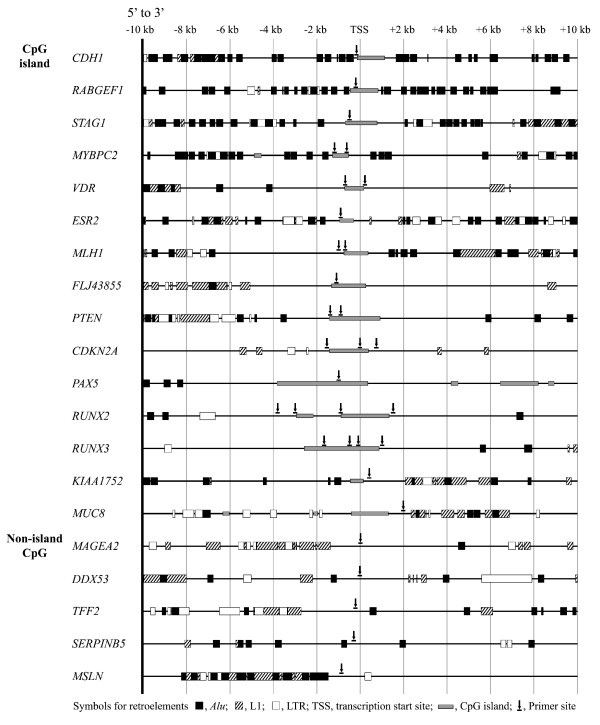
**Schematic diagram of CpG islands and retroelement distributions in the 5'-end regions of 20 genes**. A total of 32 CpG amplicon sites from 20 genes were examined using the methylation-specific PCR (MSP) primer sets. The CpG islands of the *CDH1*, *RABGEF1*, *STAG1*, and *MYBPC2 *genes were neighbored by the high-density *Alu *elements at distances of ≤ 1 kb. The *VDR*, *ESR2*, *MLH1*, *FLJ43855*, *MYBPC2*, *PTEN*, and *CDKN2A *genes confronted by a low density of *Alu*, L1, and LTR elements at a distance of 2–6 kb were examined at the boundary of CpG islands. The *PAX5*, *RUNX2*, and *RUNX3 *genes contained long CpG islands bordered by a few retroelements at distances of > 6 kb. The genes containing no CpG islands are confronted by the L1 elements (*MAGEA2 *and *TFF2*), a low density of *Alu *elements (*SERPINB5 *and *DDX53*), or a high density of *Alu *and L1 elements (*MSLN*). The *VDR*, *CDKN2A*, *RUNX2*, *RUNX3*, *KIAA1752*, and *MUC8 *genes were examined at the intragenic CpG sites of the CpG islands as well. The MSP primer sites were located in close proximity to the transcription start sites without CpG islands.

The semiquantitative methylation analysis using a radioisotope was performed in a minimum of amplification rounds for sub-plateau DNA amplification, as described elsewhere [20]. A 1 μl aliquot of the bisulfite-modified DNA was amplified and labeled in 10 μl of a hot-start PCR solution containing an α-^32^P dTTP (PerkinElmer, Boston, MA, USA) and dNTP mixture through 32 PCR cycles. Thirty-two MSP primer sets were selected at the 5'-end regions of 20 genes using the MethPrimer software [28] (table 1). The sequences, PCR condition, and genomic position of these primer set are listed in table 2 and [see Additional file 1]. The coverage of CpG islands, the distribution of retrolements, and the MSP primer position are shown in the figure 2. Each MSP primer set was designed to generate a small fragment of ≤ 150 bp, and yield a specific PCR intensity using the genomic DNA obtained from the formalin-fixed paraffin-embedded tissues.

**Figure 3:**
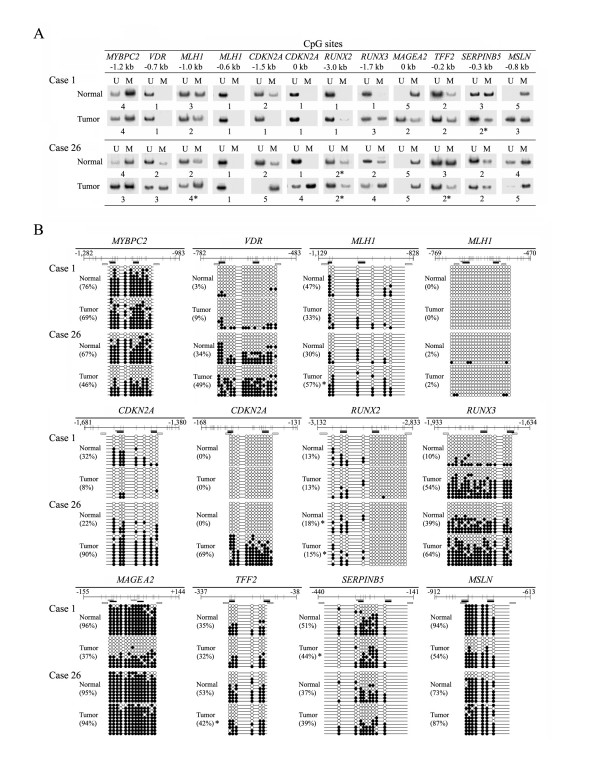
**Representative autoradiographs of semiquantitative methylation analysis (A) and the number and position of the methylated CpGs identified by the sequencing of common PCR DNA (B)**. (A) The 5'-transitional CpG sites generated a wide range of methylated and unmethylated PCR band intensities from the bisulfite-modified DNA from Case 1, with a high-level chromosomal loss, and Case 26, with a low-level chromosomal loss. The lanes marked U and M indicate the PCR bands of the unmethylation and methylation primer sets, respectively. The level of the methylated CpGs calculated from a standard MSP curve is indicated below the lanes. (B) For the same cases, the content of methylated CpGs was estimated from the common PCR DNA containing both the methylated and unmethylated CpGs. Ten common PCR clones per DNA sample were sequenced. The content of methylated CpGs is indicated as a percentage on the left side of each CpG methylation map. The different methylation results between the MSP bands and the common PCR DNAs are indicated by an asterisk.

The specificity of each MSP primer set was validated using a standard curve for the universal methylated and unmethylated DNA (figure 1B). Based on the standard curve of the control MSP bands, the density of the methylated CpGs was classified into 5 levels; level 1 (0–20% methylation), level 2 (21–40% methylation), level 3 (41–60% methylation), level 4 (61–80% methylation), and level 5 (81–100% methylation). The intensity of the MSP bands (figure 3A) was compared with the methylated CpG content that was estimated by sequencing the common PCR DNA (figure 3B). The methylation levels estimated by the MSP intensities were consistent with the methylation densities measured from the common PCR DNAs in 23 normal (96%) and 20 tumor (83%) DNAs of 24 normal and tumor DNA pairs (table 3).

In previous studies [29,30], the MSP bands visualized by the ethidium bromide staining were classified into three methylation levels (no, weak, and strong methylation). Forty eight DNA specimens were amplified through 37 PCR cycles, of which six (13%) failed to generate any visible PCR bands by ethidium bromide staining (table 3). Eight of the 42 visible MSP bands (19%) were scored differently from the common PCR DNA. Twelve of the 14 DNA specimens (85%) showing no visible PCR bands or inaccurate methylation levels were similarly scored by both 32-cycle amplification using a radioisotope and common PCR DNA. This high accuracy of the 32-cycle amplification protocol allowed the MSP bands to be classified into five methylation levels.

When 80 normal and tumor DNA pairs showing a one-level methylation difference were analyzed, 71 pairs (89%) demonstrated same-level methylation differences in the MSP band intensities and the common PCR DNA [see Additional file 2].

### Statistical analysis

The Pearson's correlation coefficient was used to determine the correlation between (i) the MSP band intensity and the proportion of methylated and unmethylated control DNA, (ii) the coverage of CpG islands and the density of retroelements, and (iii) the frequency of methylation alterations and the level of LOHs using SPSS ver. 11.0 software. A *χ*^*2 *^test or Fisher's exact test and an independent *t *test were used to compare the clinicopathological variables or the frequency of methylation alterations between the different microsatellite genotypes. Two-sided p values < 0.05 were considered significant.

## Results

### Analysis of the transitional area between the CpG Islands and the nearest retroelements

**Figure 4:**
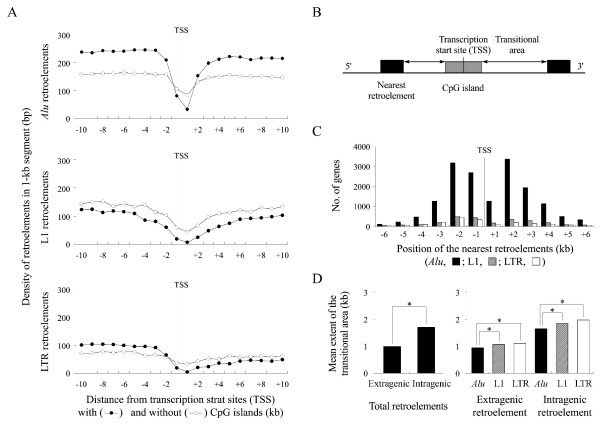
**Distributions of the *Alu*, L1, and LTR retroelements in the 5'-end regions of the human genes**. (A) The proportion of *Alu*, L1, and LTR elements in a 1-kb nucleotide bin were plotted separately in both extragenic and intragenic 10-kb segments. (B) The transitional area is demarcated by the distance between the 5'(3')-end of a CpG island and the extragenic (intragenic) nearest retroelements. The number of genes (C) and the mean extent of the transitional area (D) were calculated according the position and type of the nearest retroelements.

Of a total of 22,297 genes examined, 10,515 and 11,782 were found to have a single CpG island and no CpG islands at the transcription start site, respectively. The densities of *Alu*s, L1s, and LTRs in a 1-kb window were plotted separately a distance 10 kb upstream and downstream from the transcription start sites. Figure 4A revealed the following: (i) the densities of the three retroelement types were commonly higher in the extragenic 10-kb regions than in the 10-kb intragenic regions. (ii) the three retroelement types were commonly depressed in both the intragenic and extragenic first 1-kb regions, (iii) the density of depressed retroelements was lower in the first 1-kb regions containing CpG islands than in the regions containing no CpG islands, (iv) the density of *Alu *copies rapidly reached a plateau at a distance of 3 kb, which was higher in the 5'-end regions containing CpG islands than in the regions containing no CpG islands.

A transitional area between the CpG islands and their nearest retroelements was demarcated by a distance from the 5'(3')-end of the CpG island to the position of the nearest retroelement in the extragenic (intragenic) region (figure 4B). Figure 4C shows that the CpG islands frequently neighbored the nearest *Alu *elements (76% and 83%) and infrequently the nearest L1 (13% and 11%) and LTR (11% and 6%) elements in both the extragenic and intragenic regions. Figure 4D shows that the mean extent of the transitional area between the CpG island and the nearest retroelement was significantly shorter in the extragenic regions (989 bp) than in the intragenic regions (1,700 bp). The mean extent of the extragenic and intragenic transitional area bordered by the nearest *Alu *elements (945 bp and 1,645 bp) was significantly shorter than that by the nearest L1 (1,063 bp and 1,853 bp) and LTR (1,104 bp and 1,967 bp) elements.

### Genetic classification of gastric cancers according to the level of LOHs

The allelic profiles of the 40 microsatellite sequences were initially analyzed for a MSI at the homozygous markers that showed a few shadow or stutter bands in a pair of normal and tumor DNAs. Five of 50 gastric cancers examined had a high-frequency MSI in more than 40% of the homozygous alleles examined. A LOH was analyzed at the stable heterozygous alleles in the remaining 45 MSI-negative gastric cancers. The number of chromosomes showing more than one LOH event was counted in each MSI-negative case. Using the number of chromosomal losses, the extents of LOH was categorized into high level involving four or more chromosomes (LOH-H) and low level involving less than four chromosomes (LOH-L) for intestinal-type cancers. One and zero chromosomal loss were scored as baseline-level loss (LOH-B) for diffuse-type cancers. Overall, 20 LOH-H (40%), 19 LOH-L (38%), 6 LOH-B (12%), and 5 MSI (10%) cases were identified in the 50 surgical specimens (table 4). A comparison of the clinicopathological variables in the LOH-H and LOH-L gastric cancers revealed a lymphatic invasion (p = 0.001) and advanced stage (p = 0.015) to be significantly associated with the LOH-H cases. Intestinal-type cancers (p = 0.032) as well as well and moderate differentiation (p = 0.046) were more frequent in the LOH-L cases.

### Relationships between the LOHs and methylation alterations

**Figure 5:**
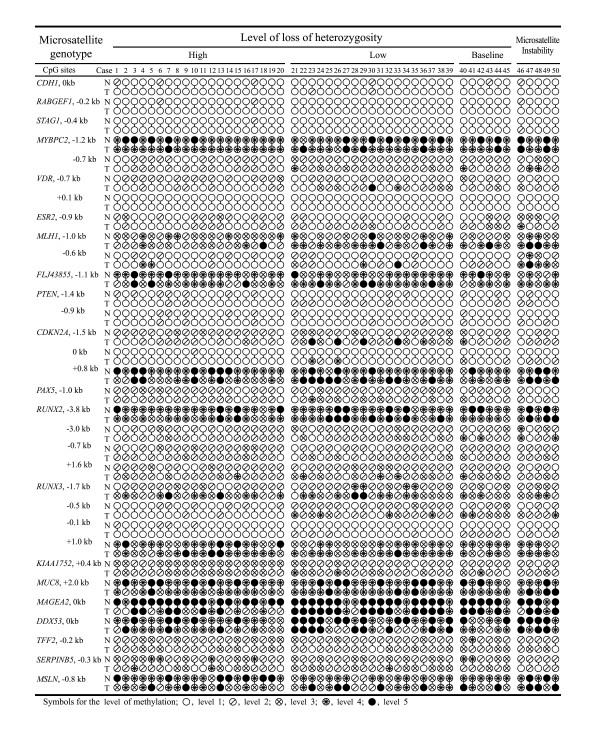
**Methylation profiles of the CpG islands and the 5'-transitional CpGs examined in the normal (N) and tumor (T) tissues of gastric cancer patients using semiquantitative methylation-specific PCR analysis**. The methylation status was classified into 5 levels. Gastric cancers were classified into four microsatellite genotypes. The level of methylation and the classification of the microsatellite genotypes are detailed in the "Materials and Methods" section. All the information regarding the methylation analysis is listed in table 1 and 2. CpG sites are indicated by the name of the gene and the distance from the transcription start site.

Fifty pairs of normal and tumor tissues were examined using the MSP primer sets for a total of 32 CpG amplicon sites (figure 5). The CpG islands of the *CDH1*, *RABGEF1*, and *STAG1 *genes, which neighbored a high density of *Alu *elements at a distance < 2 kb, were found to be hypomethylated in the normal tissues at a level < 2. These CpG islands showed few methylation alterations in the tumor tissues. The transitional CpGs of the *MYBPC2 *gene bordered by the high-density *Alus *were hypermethylated at a level ≥ 3 in the normal tissues but were unchanged in the tumor tissues. The long CpG islands of the *PAX5 *and *RUNX2 *genes neighboring a small number of retroelements at a long distance, > 6 kb, showed insignificant methylation alterations in the tumor tissues.

The CpG island boundaries neighboring a low density of *Alu *elements or L1 and LTR elements at a distance of 2–6 kb became increasingly methylated at a level of ≥ 2. The CpG sites in close proximity to the transcription start sites with no accompanying CpG islands were densely methylated at a level of ≥ 3. The CpG island boundaries and the proximal sites of the non-CpG-island genes were categorized as transitional CpGs, because these CpG sites could be distinguished from the proximal sites of the CpG islands that are consistently unmethylated. The methylation level of these 5'-transitional CpGs in the normal and tumor DNA was compared (table 5). The methylation level of the LOH-H cancers was significantly lower at the 5'-transitional CpG sites of the *VDR*, *FLJ43855*, *CDKN2A*, *MUC8*, *MAGEA2*, and *MSLN *genes. The LOH-L cancers had a significantly higher methylation level at the 5'-transitional CpG sites of the *MLH1 *and *CDKN2A *genes.

**Figure 6:**
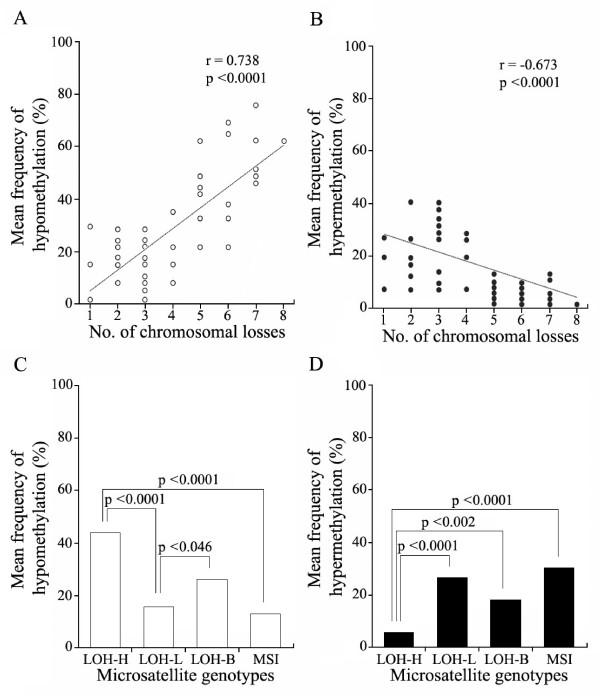
**Relationships between methylation alterations and the number of chromosomal losses in gastric cancers**. The frequency of hypomethylation (A) and hypermethylation (B) detected in 14 transitional CpG amplicons was plotted as a function of the chromosomal losses. The correlation coefficients were calculated using Pearson's correlation analysis. The correlation between methylation alterations and chromosomal losses is shown with the line of best fit. The mean frequency of hypomethylation (C) and hypermethylation (D) was compared among four microsatellite genotypes.

The frequency of methylation alterations occurring in a total of 14 transitional CpG amplicons was scored according to the number of the amplicons showing a difference in the level of methylation between the normal and tumor DNA. The frequency of hypomethylation and hypermethylation in the transitional CpGs were linearly (r = 0.738, p < 0.0001) and inversely (r = 0.673, p < 0.0001) proportional to the number of chromosomal losses, respectively, (figure 6A and 6B). There were significant differences in the mean frequency of hypomethylation (44% versus 15%) and hypermethylation (5% versus 26%) between the LOH-H and LOH-L cancers (p < 0.0001) (figure 6C and 6D). The frequency of hypermethylation in the transitional CpGs was highest in the MSI cases.

Although the proximal portion of the CpG islands was largely unmethylated in both the normal and tumor DNAs, the CpG islands of the *MLH1 *and *RUNX3 *genes was hypermethylated in most tumor tissues with a MSI (*MLH1*) and LOH-B (*RUNX3*), respectively (figure 5). The distal portion of the *RUNX3 *CpG island was frequently hypermethylated in both LOH-H and LOH-L cases.

### Relationships between methylation alterations and the clinicopathological features

The relationship between the frequency of methylation alterations and the clinicopathological features was analyzed in 39 gastric cancers with high-level and low-level chromosomal losses (table 6). The frequency of hypomethylation in the transitional area increased with increasing lymphatic invasion (p = 0.006) and advanced tumor stage (p = 0.015). The frequency of hypermethylation decreased with increasing venous invasion (p = 0.041), lymphatic invasion (p = 0.003), and advanced tumor stage (p = 0.023).

Of a total of 598 methylation alterations, 472 (79%) showed a one-level difference in methylation between the paired normal and tumor DNA and 126 (21%) showed a two- or higher-level difference with an inverse methylation status between the normal and tumor DNA [see Additional file 2]. The normal tissues of the LOH-H and LOH-L cases showed a similar level of methylation in the transitional area.

## Discussion

It was previously reported that the short-distance spread of *Alu *methylation acts as a barrier blocking the long-distance spread of L1 methylation [16]. In this study, the transitional area bordered by *Alu *elements was significantly shorter than that bordered by the L1 elements, which concurs with a previous report [16]. CpG islands neighboring the high-density *Alu *elements were invariably hypomethylated in both the normal and tumor tissues. The long CpG islands of the *PAX5 *and *RUNX2 *genes that neighbored a few retroelements showed insignificant methylation alterations. The 5'-transitional methylated CpGs bordered by either low-density *Alu *elements or other retroelements were frequently hypomethylated in the LOH-H cases (table 5). This suggests that LOH events have no influence on the unmethylated CpG islands that are protected by high-density *Alu *elements. In contrast, the transitional methylated CpG sites under long-distance L1 methylation which are either not protected or only partially protected by the *Alu *elements, were hypomethylated in association with the LOHs.

In a *Drosophila *model, repetitive RNA is believed to diffuse through the nucleus and assemble onto other homologous copies to initiate the spread of the heterochromatin signal [31]. This study found that the intragenic regions contained low-density retroelements compared with the extragenic regions, whereas the transitional area was longer in the intragenic regions than in the extragenic regions (figure 4D). The intragenic retroelements releasing RNA, expressed in initial embryogenesis, are believed to have a higher probability for encountering homologous RNA sequences to promote the further spread of methylation compared with extragenic copies releasing fewer RNA. Therefore, the 5'-transitional CpG sites affected by the RNA-mediated spread of L1 methylation are likely to be hypomethylated as a result of chromosomal losses.

The hypomethylation of imprinted genes is often observed in gastric cancers but not associated with a LOH involving the counterpart chromosome [32,33]. The losses of chromosome 9p (*CDKN2A*) and 3p (*MLH1*) examined in a previous study [20] were not associated with the hypomethylation of the transitional CpGs of the *CDKN2A *and *MLH1 *genes. This suggests that epigenetic alterations in response to the LOH events are more likely to result from genome-wide compensation than chromosome by chromosome compensation. Meanwhile, a previous study on colonic cancers reported that the LOH events examined on three chromosomes were associated with global L1 hypomethylation [19]. In this study on gastric cancers, there is a significant difference in L1-associated methylation between the LOH-H and LOH-L cases. Therefore, the reduced spread of methylation in the transitional area is likely due to LOH events accompanying an unbalanced decrease in the genomic L1 dose.

Previous multifocal LOH studies [6,27,34,35] reported that heterogeneous tumor sites shared a similar level of LOHs involving the same and different chromosomes. Interestingly, heterogeneous tumor sites of multidirectionally differentiated cancers such as a glandular-neuroendocrine carcinoma [34], sarcomatoid carcinoma [35], and gastric cancers [6,20,27] frequently demonstrated reciprocal losses involving the opposite alleles on the same chromosome. This suggests that a similar level of LOHs rather than the genomic position drives the subclonal expansion of heterogeneous tumor cells with a similar growth advantage, and that the level of LOHs acts as a stem-line genotype driving tumor progression.

A previous report on multifocal methylation analysis [20] showed that the methylation alterations associated with the level of LOHs were scored differentially in heterogeneous sites from a given gastric cancer. This suggests that chromosomal losses cause sideline methylation alterations and initiate intratumoral methylation heterogeneity. The transitional CpG sites of individual genes mostly demonstrated a one-level difference in the methylation status between normal and tumor tissues [see Additional file 2]. Bisulfite sequencing analyses using the tumor cell lines and pure primary tissues revealed low-density methylation in the unmethylated CpG islands [36-39]. However, the high tumor cell content examined in this study unambiguously demonstrated LOH events. Therefore, low-level methylation changes are the result of methylation heterogeneity rather than the low tumor cell content.

The hypomethylation of the 5'-transitional CpG sites was associated with the clinicopathological variables in terms of the similarities to the LOH-H cases (tables 4 and 6). The LOH-H cases have been reported to be associated with a poor prognosis [4,6,27]. The poor clinical course of gastric cancer might be the result of an interaction between stem-line high-level LOHs and sideline hypomethylation. Considering that the 5'-transitional CpG sites were methylated to various degrees in a tissue-specific manner [16], the hypomethylation of the transitional CpGs may play a role in the malignant cellular transformation. Extra-embryonic trophoectodermal cells highlight the invasive potential of hypomethylated cells, which penetrate the endometrium during early embryogenesis and implantation. A number of studies have reported hypomethylated trophoblast cells to be well equipped with the biochemical mediators essential for invasive growth and metastasis in a similar manner to transformed tumor cells [40]. Therefore, it appears that the transformation, invasion, and metastasis of tumor cells is dynamically stimulated by the stochastic hypomethylation in response to LOH events rather than by the simple up-regulation of gene expression.

The CpG island of the *RUNX3 *gene is frequently hypermethylated in gastric cancer [41]. In this study, the CpG island of the *RUNX3 *gene was specifically methylated in the LOH-B gastric cancers (figure 5), which were associated with diffuse-type cancers without forming well-defined glandular structures (table 4). The 5'-transitional CpGs of genes other than *RUNX3 *were more frequently hypomethylated in the LOH-B cases than in the LOH-L and MSI cases (figure 6C). The *RUNX3 *gene plays a key role in controlling many of the genes associated with the differentiation of gastric epithelial cells [42]. The hypermethylated CpG island of the *RUNX3 *gene might be responsible for extensive gene inactivation in gastric epithelial cells developing into diffuse-type cancers. Accordingly, silencing of such master genes should cause widespread alterations in the intronic retroelement transcripts and increase the hypomethylation changes in the transitional area.

The CpG island of the *MLH1 *gene was specifically hypermethylated in the MSI cases, in which the hypermethylation of the 5'-transitional CpGs was most frequent (figure 6D). This is in agreement with previous studies reporting that the MSI-positive gastrointestinal cancers accompany the frequent hypermethylation of CpG islands [38,43]. MSI-associated hypermethylation is frequently observed in the intestinal metaplasia and adenoma [36,39], and the MSI cases has been reported to have a favourable clinical outcome even in large-sized tumors [6,27]. In this study, the 5'-transitional CpGs were infrequently hypermethylated in gastric cancers with a lymphatic and venous invasion and an advanced stage (table 6). It is likely that hypermethylation of the transitional CpGs can play more of a role in the initiation of a gastric cancer [44], rather than its progression.

## Conclusion

A dosage compensation mechanism is believed to maintain a balance in the dose of L1 elements, which initiate the long-distance spread of methylation involving the genome-wide as well as transitional CpGs. The transitional methylated CpG sites not completely protected by the *Alu *elements were hypomethylated in association with LOH events in gastric cancers. This suggests that an irreversible unbalanced decrease in the genomic dose reduces the spread of L1 methylation in the 5'-end regions of genes in gastric cancers.

## Competing interests

The author(s) declare that they have no competing interests.

## Authors' contributions

YHK: Performed the MSP experiments and involved in paper writing

SJH: performed DNA modification and cloning sequencing

YCJ: performed human genome data analysis

SJK: performed LOH analysis

EJS: helped to histopathologic analysis

SWC: contributed to clinical sample collection

MGR: 1) Designed MSP experiment, human genome data analysis, and LOH experiment, and interpreted all experimental and *in silico *data; 2) Wrote the initially submitted manuscript, resubmitted version, and revised version; and 3) have given final approval of the version to be published.

All authors read and approved the final manuscript.

## Pre-publication history

The pre-publication history for this paper can be accessed here:



## Supplementary Material

Additional file 1 **Sequences and PCR condition of unmethylation (U) and methylation (M) specific primer sets**. CpG sites are indicated by the name of the gene and the distance from the transcription start site. Click here for file

Additional file 2 **Methylation alterations detected in 50 gastric cancers by methylation-specific PCR analysis and sequencing of common PCR DNA**. CpG sites are indicated by the name of the gene and the distance from the transcription start site. One-level methylation alterations were analysed by the methylation-specific PCR intensity and the common PCR DNA. Click here for file

## Figures and Tables

**Table 1:** Summary of 20 genes examined by methylation analysis

Gene symbol	Synonym	Gene description	Accession no.
CpG islands			
*CDH1*	*E-CADHERIN*	Cadherin 1, type 1, E-cadherin (epithelial)	NM_004360
*RABGEF1*	*AJ250042*	RAB guanine nucleotide exchange factor (GEF) 1	NM_014504
*STAG1*	*SA1*	Stromal antigen 1	NM_005862
*MYBPC2*	*AJ250042*	Myosin binding protein C, fast type	NM_004533
*VDR*	*J03258*	Vitamin D (1,25- dihydroxyvitamin D3) receptor	NM_000376
*ESR2*	*AF051428*	Estrogen receptor 2 (ER beta)	NM_001437
*MLH1*	*HMLH1*	MutL homolog 1, colon cancer, nonpolyposis type 2	NM_000249
*FLJ43855*	*AK125843*	similar to sodium- and chloride-dependent creatine transporter	NM_198857
*PTEN*	*BZS*	Phosphatase and tensin homolog	NM_000314
*CDKN2A*	*P16*	Cyclin-dependent kinase inhibitor 2A	NM_000077
*PAX5*	*AY463952*	Paired box gene 5 (B-cell lineage specific activator)	NM_016734
*RUNX2*	*CBFA1*	Runt-related transcription factor 2	NM_004348
*RUNX3*	*CBFA3*	Runt-related transcription factor 3	NM_004350
*KIAA1752*		Homo sapiens mRNA for KIAA1752 protein	AB051539*
*MUC8*		Mucin 8, tracheobronchial	U14383*
Non-island CpGs			
*MAGEA2*	*MAGE-A2*	Melanoma antigen family A, 2	NM_005361
*DDX53*	*CAGE*	DEAD (Asp-Glu-Ala-Asp) box polypeptide 53	NM_182699
*TFF2*	*SML1*	Trefoil factor 2 (spasmolytic protein 1)	NM_005423
*SERPINB5*	*MASPIN*	Serpin peptidase inhibitor, clade B (ovalbumin), member 5	NM_002639
*MSLN*	*MPF*	Mesothelin isoform 2 precursor	NM_013404

*GenBank accession number.

**Table 2:** CpG dinucleotides examined by methylation-specific polymerase chain reaction

					Primer position*		Intensity ratio†		
						
Gene	Chromosome locus	CpG sites (kb)*	CpG ratio	GC%	U	M	U	U/M‡	M
*CDH1*	16q22	0	0.73	70	-78	-76	0.92	0.49/0.51	0.94
*RABGEF1*	7q11	-0.2	1.00	67	-285	-283	0.9	0.46/0.54	0.91
*STAG1*	3q22	-0.4	0.95	62	-493	-490	0.93	0.51/0.49	0.91
*MYBPC2*	19q13	-1.2	0.61	61	-1,226	-1,224	0.92	0.48/0.52	0.94
		-0.6	0.83	67	-742	-742	0.9	0.51/0.49	0.88
*VDR*	12q13	-0.7	0.71	58	-728	-727	0.92	0.51/0.49	0.92
		+0.1	0.84	63	+45	+45	0.95	0.51/0.49	0.94
*ESR2*	14q23	-0.9	0.66	59	-983	-986	0.85	0.48/0.52	0.91
*MLH1*	3p22	-1.0	0.43	49	-1,098	-1,098	0.93	0.51/0.49	0.95
		-0.6	0.85	63	-660	-655	0.94	0.48/0.52	0.93
*FLJ43855*	16p11	-1.1	0.62	55	-1147	-1147	0.89	0.48/0.52	0.90
*PTEN*	10q23	-1.4	0.66	62	-1,425	-1,425	0.93	0.52/0.48	0.91
		-0.9	0.86	62	-953	-953	0.94	0.51/0.49	0.93
*CDKN2A*	9p21	-1.5	0.77	65	-1,580	-1,575	0.9	0.47/0.53	0.93
		0	1.10	65	-85	-85	0.91	0.47/0.53	0.91
		+0.8	0.48	51	776	775	0.93	0.51/0.49	0.96
*PAX5*	9p13	-1.0	0.50	63	-1,056	-1,054	0.92	0.52/0.48	0.90
*RUNX2*	6p21	-3.8	0.25	36	-3,883	-3,883	0.9	0.48/0.52	0.93
		-3.0	0.71	55	-3,061	-3,061	0.91	0.49/0.51	0.92
		-0.7	0.81	62	-852	-850	0.93	0.52/0.48	0.92
		+1.6	0.63	53	1573	1573	0.92	0.47/0.53	0.94
*RUNX3*	1p36	-1.7	0.82	60	-1,790	-1,790	0.89	0.46/0.54	0.9
		-0.5	0.94	75	-559	-559	0.91	0.48/0.52	0.9
		-0.1	1.07	76	-219	-217	0.92	0.5/0.5	0.93
		+1.0	0.79	59	952	952	0.91	0.49/0.51	0.93
*KIAA1752*	16q12	+0.4	0.42	41	391	391	0.92	0.48/0.52	0.94
*MUC8*	12q24	+2.0	0.42	59	1,909	1,909	0.94	0.49/0.51	0.95
*MAGEA2*	Xq28	0	0.56	64	-81	-75	0.9	0.54/0.46	0.91
*DDX53*	Xq22.11	0	0.53	59	-71	-102	0.94	0.48/0.52	0.94
*TFF2*	21q22	-0.2	0.32	57	-251	-251	0.96	0.5/0.5	0.96
*SERPINB5*	18q21	-0.3	0.62	51	-315	-308	0.93	0.51/0.49	0.94
*MSLN*	16q13	-0.8	0.52	57	-827	-827	0.92	0.47/0.53	0.93

*The genomic position was calculated from the May 2004 human reference sequence.

†The relative proportion of unmethylation (U) and methylation (M) band intensity against the total band intensity.

‡U/M indicates a PCR mixture containing the same amount of the universal methylated and unmethylated DNA.

**Table 3:** The density of methylation estimated by semiquantitative methylation-specific PCR analysis and sequencing of common PCR DNA

			Methylation-specific PCR		
				
CpG sites*	Case no.		32-cycle amplicons using radioisope	37-cycle amplicons using ethidium bromide	% Methylation in 10 common PCR clones
*MYBPC2*, -1.2 kb	1	Normal	Level 4	Level 4	53/70‡ (76%)
		Tumor	Level 4	Level 4	48/70 (69%)
	26	Normal	Level 4	Level 4	47/70 (67%)
		Tumor	Level 3	No band	32/70 (46%)
*VDR*, -0.7 kb	1	Normal	Level 1	Level 1	4/130 (3%)
		Tumor	Level 1	Level 1	12/130 (9%)
	26	Normal	Level 2	Level 2	44/130 (34%)
		Tumor	Level 3	Level 3	64/130 (49%)
*MLH1*, -1.0 kb	1	Normal	Level 3	Level 3	14/30 (47%)
		Tumor	Level 2	Level 3†	10/30 (33%)
	26	Normal	Level 2	No band	9/30 (30%)
		Tumor	Level 4†	Level 3	17/30 (57%)
*MLH1*, -0.6 kb	1	Normal	Level 1	Level 1	0/200 (0%)
		Tumor	Level 1	Level 1	0/200 (0%)
	26	Normal	Level 1	Level 1	4/200 (2%)
		Tumor	Level 1	Level 1	4/200 (2%)
*CDKN2A*, -1.5 kb	1	Normal	Level 2	Level 2	19/60 (32%)
		Tumor	Level 1	Level 1	5/60 (8%)
	26	Normal	Level 2	Level 2	13/60 (22%)
		Tumor	Level 5	No band	54/60 (90%)
*CDKN2A*, 0 kb	1	Normal	Level 1	Level 1	0/110 (0%)
		Tumor	Level 1	Level 1	0/110 (0%)
	26	Normal	Level 1	Level 1	0/110 (0%)
		Tumor	Level 4	Level 3†	76/110 (69%)
*RUNX2*, -3.0 kb	1	Normal	Level 1	Level 1	8/60 (13%)
		Tumor	Level 1	Level 1	8/60 (13%)
	26	Normal	Level 2†	No band	11/60 (18%)
		Tumor	Level 2†	Level 1	9/60 (15%)
*RUNX3*, -1.7 kb	1	Normal	Level 1	Level 1	6/70 (10%)
		Tumor	Level 3	Level 3	38/70 (54%)
	26	Normal	Level 2	Level 3†	27/70 (39%)
		Tumor	Level 4	Level 3†	45/70 (64%)
*MAGEA2*, 0 kb	1	Normal	Level 5	Level 5	106/110 (96%)
		Tumor	Level 2	Level 2	41/110 (37%)
	26	Normal	Level 5	Level 5	104/110 (95%)
		Tumor	Level 5	Level 5	103/110 (94%)
*TFF2*, -0.2 kb	1	Normal	Level 2	Level 3†	21/60 (35%)
		Tumor	Level 2	Level 3†	19/60 (32%)
	26	Normal	Level 3	Level 3	32/60 (53%)
		Tumor	Level 2†	Level 3	25/60 (42%)
*SERPINB5*, -0.3 kb	1	Normal	Level 3	Level 3	36/70 (51%)
		Tumor	Level 2†	No band	31/70 (44%)
	26	Normal	Level 2	Level 3†	26/70 (37%)
		Tumor	Level 2	No band	27/70 (39%)
*MSLN*, -0.8 kb	1	Normal	Level 5	Level 5	66/70 (94%)
		Tumor	Level 3	Level 4†	38/70 (54%)
	26	Normal	Level 4	Level 4	51/70 (73%)
		Tumor	Level 5	Level 5	61/70 (87%)

*CpG sites are indicated by the name of the gene and the distance from the transcription start site.

†Different results between methylation-specific PCR and common PCR DNA.

‡'/' separates the number of the methylated CpGs and the total CpGs.

**Table 4:** Clinicopathological characteristics of gastric cancers according to the level of loss of heterozygosity (LOH) and microsatellite instability*

	Total	High LOH	Low LOH	p†	Baseline LOH	Microsatellite instability
No. of patients	50	20	19		6	5
Age (years)						
Mean ± SD	59.6 ± 12.6	60.5 ± 11.7	61.1 ± 11.6	0.883	55.0 ± 7.9	66.8 ± 7.3
Tumor size (cm)						
Mean ± SD	4.9 ± 2.8	5.2 ± 2.7	4.4 ± 3.1	0.402	4.3 ± 1.8	6.3 ± 3.2
Sex						
Male	32	15	12	0.325	3	2
Female	18	5	7		3	3
Lauren classification						
Intestinal	29	10	16	0.032	0	3
Diffuse	11	5	3		3	0
Mixed	10	5	0		3	2
Differentiation						
Well	4	0	3	0.046	0	1
Moderate	26	11	13		0	2
Poor	20	9	3		6	2
Growth pattern						
Infiltrative	27	14	9	0.126	2	2
Expanding	3	0	3		0	0
Mixed	20	6	7		4	3
Venous invasion						
Yes	6	4	0	0.059	2	0
No	44	16	19		4	5
Lymphatic invasion						
Yes	29	16	5	0.001	5	3
No	21	4	14		1	2
Tumor stage						
Early stage	29	8	15	0.015	2	4
Advanced stage	21	12	4		4	1

*The classification of microsatellite genotypes is detailed in the "Material and Method" section.

†p values were calculated between the low LOH and high LOH cases by an independent *t *test for the age and tumor size variables and by a Fisher's exact test or a *χ*^*2 *^test for other variables.

**Table 5:** Methylation alterations in the CpG islands and the 5'-transitional CpG sites examined in gastric cancers containing high-level and low-level chromosomal losses

	High-level chromosomal loss (n = 20)					Low-level chromosomal loss (n = 19)				
		
	Mean level of methylation			Methylation alterations (%)		Mean level of methylation			Methylation alterations (%)	
						
CpG sites*	Normal	Tumor	p†	Hypo-	Hyper-	Normal	Tumor	p†	Hypo-	Hyper-
CpG islands										
*CDH1*, 0 kb	1.2	1.0	0.083	3 (15)	0 (0)	1.1	1.1	0.560	0 (0)	1 (5)
*RABGEF1*, -0.2 kb	1.1	1.0	0.163	2 (10)	0 (0)	1.0	1.0	1	0 (0)	0 (0)
*STAG1*, -0.4 kb	1.1	1.0	0.330	1 (5)	0 (0)	1.0	1.0	1	0 (0)	0 (0)
*MYBPC2*, -0.6 kb	1.6	1.6	0.757	2 (10)	1 (5)	2.0	2.3	0.090	0 (0)	4 (21)
*MLH1*, -0.6 kb	1.4	1.4	1	5 (25)	2 (10)	1.3	1.7	0.153	0 (0)	4 (21)
*PTEN*, -0.9 kb	1.3	1.2	0.442	2 (10)	0 (0)	1.1	1.1	0.560	1 (5)	2 (11)
*CDKN2A*, 0kb	1.1	1.0	0.330	1 (5)	0 (0)	1.0	1.4	0.072	0 (0)	4 (21)
*PAX5*, -1.0 kb	2.0	1.7	0.115	7 (35)	0 (0)	1.9	2.1	0.505	4 (21)	5 (26)
*RUNX2*, -3.8 kb	4.2	3.8	0.054	7 (35)	0 (0)	4.2	4.3	0.376	1 (5)	4 (21)
-3.0 kb	1.8	1.8	1	5 (25)	5 (25)	2.0	2.0	1	3 (16)	4 (21)
-0.7 kb	2.1	2.0	0.575	5 (25)	4 (20)	1.7	1.9	0.266	4 (21)	6 (32)
+1.6 kb	2.0	2.2	0.295	3 (15)	5 (25)	2.1	2.5	0.066	2 (11)	7 (37)
*RUNX3*, -1.7 kb	2.0	3.3	<0.0001	0 (0)	14 (70)	2.5	3.3	0.012	0 (0)	12 (63)
-0.5 kb	1.3	1.1	0.121	6 (30)	2 (10)	1.8	2.4	0.035	2 (11)	9 (47)
-0.1 kb	1.2	1.0	0.042	4 (20)	0 (0)	1.0	1.2	0.042	0 (0)	4 (21)
+1.0 kb	4.0	3.8	0.519	6 (30)	5 (25)	3.4	3.8	0.012	0 (0)	7 (37)
*VDR*, +0.1 kb	1.0	1.0	1	0 (0)	0 (0)	1.0	1.0	1	0 (0)	0 (0)
*KIAA1752*, +0.4 kb	2.8	2.6	0.096	8 (40)	0 (0)	2.1	2.5	0.084	0 (0)	7 (37)
Transitional CpGs										
*MYBPC2*, -1.2 kb	4.3	4.1	0.083	4 (20)	0 (0)	4.3	4.2	0.546	5 (26)	2 (11)
*VDR*, -0.7 kb	1.8	1.3	0.003	10 (50)	0 (0)	1.5	2.1	0.056	3 (16)	8 (42)
*ESR2*, -0.9 kb	1.7	1.4	0.114	6 (30)	0 (0)	1.3	1.5	0.141	0 (0)	4 (22)
*MLH1*, -1.0 kb	3.1	2.5	0.050	9 (45)	2 (10)	2.8	3.5	0.035	1 (5)	11 (58)
*FLJ43855*, -1.1 kb	4.0	3.5	0.043	10 (50)	2 (10)	3.7	4.1	0.072	2 (11)	8 (42)
*PTEN*, -1.4 kb	1.4	1.1	0.062	5 (25)	0 (0)	1.1	1.3	0.119	1 (5)	5 (26)
*CDKN2A*, -1.5 kb	2.0	1.6	0.012	10 (50)	2 (10)	2.0	2.8	0.025	3 (16)	10 (53)
+0.8 kb	4.4	3.6	0.002	12 (60)	0 (0)	4.1	4.4	0.173	3 (16)	7 (37)
*MUC8*, +2.0 kb	4.4	3.7	0.001	9 (45)	0 (0)	4.4	4.2	0.149	6 (32)	2 (11)
*MAGEA2*, 0 kb	4.8	3.4	<0.0001	11 (55)	0 (0)	4.8	4.6	0.336	2 (11)	0 (0)
*DDX53*, 0 kb	4.2	3.7	0.051	9 (45)	2 (10)	4.5	3.9	0.096	6 (32)	2 (11)
*TFF2*, -0.2 kb	2.4	2.1	0.174	7 (35)	2 (10)	2.2	2.0	0.324	6 (32)	2 (11)
*SERPINB5*, -0.3 kb	2.7	2.2	0.080	10 (50)	3 (15)	2.4	2.5	0.598	2 (11)	4 (21)
*MSLN*, -0.8 kb	4.3	3.6	0.005	11 (55)	2 (10)	3.5	3.7	0.591	1 (5)	5 (26)

*CpG sites are indicated by the name of the gene and the distance from the transcription start site.

†p values were calculated by an independent *t *test for the mean values.

**Table 6:** Relationships between methylation alterations and clinicopathological variables in gastric cancers (n = 39) containing high-level and low-level chromosomal losses

		Mean frequency of methylation alterations* (%, mean ± SD)			
		
Clinicopathological variables	No. of patients	Hypomethylation	p†	Hypermethylation	p†
Age (years)					
≤55	7	34.7 ± 28.4	0.547	11.2 ± 9.1	0.384
>55	32	29.0 ± 20.1		16.5 ± 15.2	
Tumor size (cm)					
≤5	24	24.7 ± 16.7	0.056	17.3 ± 15.6	0.359
> 5	15	38.6 ± 27.4		12.9 ± 12.1	
Sex					
Male	27	28.8 ± 22.2	0.619	14.5 ± 15.0	0.514
Female	12	32.7 ± 22.9		17.8 ± 13.1	
Lauren classification					
Intestinal	26	28.0 ± 21.3	0.727	18.1 ± 15.0	0.189
Diffuse	8	34.8 ± 29.4		13.4 ± 12.9	
Mixed	5	32.9 ± 15.6		5.7 ± 9.3	
Differentiation					
Well	3	23.8 ± 10.9	0.671	23.8 ± 8.2	0.169
Moderate	24	28.6 ± 22.2		17.6 ± 15.3	
Poor	12	34.5 ± 24.7		9.5 ± 11.9	
Growth pattern					
Infiltrative	23	32.6 ± 25.6	0.639	13.4 ± 12.9	0.221
Expanding	3	21.4 ± 14.3		28.6 ± 14.3	
Mixed	13	27.4 ± 16.7		16.5 ± 16.1	
Venous invasion					
Yes	4	37.5 ± 16.9	0.485	1.8 ± 3.6	0.041
No	35	29.2 ± 22.7		17.1 ± 14.3	
Lymphatic invasion					
Yes	21	38.8 ± 25.2	0.006	9.5 ± 10.4	0.003
No	18	19.8 ± 12.1		22.6 ± 15.3	
Tumor stage					
Early stage	23	23.0 ± 15.1	0.015	19.9 ± 15.7	0.023
Advanced stage	16	40.2 ± 26.9		9.4 ± 9.7	

*Methylation alterations were detected in 14 transitional CpG amplicons.

†p values were calculated by an independent *t *test.
