# Supplementary material for: The 5'-end transitional CpGs between the CpG islands and retroelements are hypomethylated in association with loss of heterozygosity in gastric cancers
Source: BMC Cancer. 2006 Jul 10;6:180. doi: 10.1186/1471-2407-6-180 (PMC1552088; doi:10.1186/1471-2407-6-180)
Supplement: Additional file 1 — Sequences and PCR condition of unmethylation (U) and methylation (M) specific primer sets. CpG sites are indicated by the name of the gene and the distance from the transcription start site. [file 1471-2407-6-180-S1.doc]

**Additional file 1 -** Sequences and PCR condition of unmethylation (U) and methylation (M) specific primer sets

| CpG sites* |  | Forward (5` to 3`) | Reverse (5` to 3`) | Amplicon size (bp) | Tm (^o^C) |
| --- | --- | --- | --- | --- | --- |
| *CDH*, 0 kb | U | GGTGAATTTTTAGTTAATTAGTGGTAT | TCACAAATACTTTACAATTCCAACA | 108 | 56 |
|  | M | TGAATTTTTAGTTAATTAGCGGTAC | ACAAATACTTTACAATTCCGACG | 104 | 58 |
| *RABGEF*, 0.2kb | U | AAGTTGGAAGTAGGGATTGAGT | CAAAATAAAATACCACCCTAACA | 131 | 58 |
|  | M | GTCGGAAGTAGGGATTGAGC | GAAATAAAATACCGCCCTAACG | 128 | 58 |
| *STAG1*, -0.4 kb | U | TTTTTAGGTTTTAGGGTTGGT | ACCCTCAAATTTCCACAAAACA | 96 | 58 |
|  | M | TTTTTTAGGTTTTAGGGTCGGC | CTCGAATTTCCGCAAAACG | 94 | 58 |
| *MYBPC2*, -1.2 kb | U | TTTTTAATTTAGTGGGGTTTGT | AAAAACATCCAACCAATCCA | 96 | 58 |
|  | M | TTTAATTTAGCGGGGTTCGC | AAAAACGTCCAACCAATCCG | 94 | 60 |
| -0.6 kb | U | TGTTTGTTTTGGGAAGAGTTGT | AACTCCAAAATTTCACACCCCA | 125 | 58 |
|  | M | TGTTCGTTTCGGGAAGAGTCGC | AACTCCGAAATTTCGCGCCCCG | 125 | 60 |
| *VDR*, -0.7 kb | U | TGGTAGTGATTGTGGTTGATTAT | CCTCACACCAATACCACAAAACA | 130 | 58 |
|  | M | GGTAGCGATCGCGGTTGATTAC | CTCACGCCGATACCACGAAACG | 128 | 58 |
| +0.1 kb | U | GGTATTTTAGATGTTTTGATTTTG | AAAACAACTTATCCACCCACCAA | 102 | 58 |
|  | M | GGTATTTTAGACGTTTCGATTTCG | GAAACAACTTATCCACCCGCCGA | 102 | 58 |
| *ESR2*, -0.9 kb | U | TTTTTTTTAAGGATTTTGTGTGT | ACTAAAAATACACATTCCACCA | 111 | 56 |
|  | M | TTTTTTTAAGGATTTCGCGCGC | CCAACTAAAAATACACGTTCCACCG | 113 | 58 |
| *MLH1*, -1.0 kb | U | GATTTTAGGATTGTTGATATGAGT | AAACTACCTCCTAATCTTTATCCA | 126 | 58 |
|  | M | GATTTTAGGATTGTCGATATGAGC | AACTACCTCCTAATCTTTATCCG | 125 | 58 |
| -0.6 kb | U | TTTTGATGTAGATGTTTTATTAGGGTTGT | ACCACCTCATCATAACTACCCACA | 121 | 58 |
|  | M | ACGTAGACGTTTTATTAGGGTCGC | CCTCATCGTAACTACCCGCG | 115 | 58 |
| *FLJ43855*, -1.1 kb | U | TGGTTGTTATTTGGGGTGGTTG | CTAAACCACACTAAAAACAAACA | 111 | 56 |
|  | M | TGGTTGTTATTTGGGGCGGTC | CTAAACCACACTAAAAACGAACG | 113 | 58 |
| *PTEN*, -1.4 kb | U | TTTTGTGTTTTGTAAGAATTGGT | AACCTCCCAAAAAAACACTATCA | 124 | 58 |
|  | M | TTTCGCGTTTTGTAAGAATCGGC | ACCTCCCGAAAAAACGCTATCG | 123 | 60 |
| -0.9 kb | U | TATTTTGTTGGGTTTTTATGGT | AACTCCAAATCAATTCACAACATCA | 96 | 58 |
|  | M | TATTTTGTCGGGTTTTTACGGC | AAATCGATTCGCGACGTCG | 90 | 58 |
| *CDKN2A*, -1.5 kb | U | TTGGGATTAGGTTTAGTTTTGG | CTATAAAACCCTATCAACTCACACT | 130 | 58 |
|  | M | TCGGGATTAGGTTTAGTTTCG | AAACCCTATCGACTCACGCT | 125 | 60 |
| 0 kb | U | TGTTTATTTTTGTTTTGTAGGTG | AAAACTCAAAACCATTCCAA | 129 | 56 |
|  | M | TGTTTATTTTCGTTTCGTAGGC | AAAACTCAAAACCGTTCCGA | 129 | 58 |
| +0.8 kb | U | GTATTTTAGGAAGTTGTTGTTTGT | TTTTCTCCCCAACCTCCCAACA | 101 | 58 |
|  | M | GTATTTTAGGAAGTCGTTGTTTGC | TTTTTCTCCCCAACCTCCCGACG | 102 | 60 |
| *PAX5*, -1.0 kb | U | GTAGGAGGATTTTTGGTTTGTT | CCTAAATTACAACCCAACCTCA | 115 | 59 |
|  | M | AGGAGGATTTTTGGTTCGTC | TAAATTACGACCCAACCTCG | 111 | 59 |
| *RUNX2*, -3.8 kb | U | AGGTTTAGTTAGTTTTAGTTG | CCACTAAATACCCTAACAACA | 113 | 59 |
|  | M | AGGTTTAGTTAGTTTTAGTCG | CCACTAAATACCCTAACAACG | 113 | 59 |
| -3.0 kb | U | TGTTTGAGTGTATATGAGTGGAT | TCTCTCAAATCCCACAAACAACCA | 123 | 59 |
|  | M | TGTTCGAGTGTATATGAGTGGAC | TCTCTCGAATCCCACAAACGACCG | 123 | 59 |
| -0.7 kb | U | GGTTTTGGAAATTGTATATGGTGT | AAACAACAAATCTCAAACCTACA | 96 | 58 |
|  | M | TTTCGGAAATTGTATACGGCGC | AACAACGAATCTCGAACCTACG | 93 | 58 |
| +1.6 kb | U | GTTTGAGGGTGGGTGGTAGTTGT | ACTACCCCAAAAAATCTAAATCA | 127 | 59 |
|  | M | GTTTGAGGGCGGGTGGTAGTCGC | ACTACCCCGAAAAATCTAAATCG | 127 | 59 |
| *RUNX3*, -1.7 kb | U | TGGGGTTAGATTTTTGTTGTTTTT | ATAAAATCTTACAACCACCATCA | 107 | 56 |
|  | M | CGGGGTTAGATTTTCGTTGTTTTC | ATAAAATCTTACGACCACCGTCG | 107 | 58 |
| -0.5 kb | U | GATGTGTTGTATAGTTAATTGGT | TCCCCATTAAACAACCTCCA | 97 | 56 |
|  | M | CGCGTCGTATAGTTAATCGGC | TCCCCGTTAAACGACCTCCG | 95 | 58 |
| -0.1 kb | U | GGAAAGTAGAAGTGGTGGGGTTT | ACTAACCAAACAAACTACAAACA | 128 | 59 |
|  | M | GAAAGTAGAAGCGGCGGGGTTC | ACTAACCGAACAAACTACGAACG | 125 | 59 |
| +1.0 kb | U | GTTGTTTTAATGGGAGTAGGGAT | CAAAATAAAACAAAAACACCTCA | 147 | 59 |
|  | M | GTCGTTTTAATGGGAGTAGGGAC | GAAATAAAACGAAAACGCCTCG | 147 | 59 |
| *KIAA1752*, +0.4 kb | U | TAATGGTTTTTGAGGATTGAGATTG | CACAAACTATTATCAACCAATCACA | 103 | 58 |
|  | M | TAATGGTTTTTGAGGATTGAGATC | CACAAACTATTATCAACCGATCACG | 103 | 62 |
| *MUC8*, +2.0 kb | U | GGTAGGAGTTATTAGGAGAGTATT | AATACAAACACTCACCACCTAACCA | 140 | 55 |
|  | M | GGTAGGAGTTATTAGGAGAGTATC | AATACAAACGCTCACCGCCTAACCG | 140 | 60 |
| *MAGEA2*, 0 kb | U | GTTAGGTTGTTGTTTAGGGT | CCAAAAAAATCACAAACCCA | 92 | 59 |
|  | M | GCGTTTGTTTTTTTTCGTCGAC | AAATCACGAACCCGAATATAACG | 108 | 61 |
| *DDX53*, 0 kb | U | TGGTTTTTGGGGTAATTTTTGT | CAAATCTACAACCTATTTCCCA | 105 | 57 |
|  | M | TTTTATACGATTCGGAATTCGAC | CAAATCTACGACCTATTTCCCG | 136 | 58 |
| *TFF2*, -0.2 kb | U | GGTAGTTGTGTTTTGTGTAGGT | CACATAACCAATTTTCCACA | 130 | 56 |
|  | M | GGTAGTTGTGTTTTGTGTAGGC | CACGTAACCGATTTTCCACG | 130 | 62 |
| *SERPINB5*, -0.3 kb | U | GAATATTTTATTTTTTGGTTTTGTG | AAAAAACCTCCAACATATTCA | 111 | 56 |
|  | M | TTATTTTTCGGTTTTGCG | AAAAAACCTCCAACATATTCG | 104 | 54 |
| *MSLN*, -0.8 kb | U | GGAGAGATTAGAGATGATTGTTGT | CATAAACTCTTATCCCCAATACA | 103 | 55 |
|  | M | GGAGAGATTAGAGATGATCGTCGC | CGTAAACTCTTATCCCCAATACG | 103 | 60 |

*CpG sites are indicated by the name of the gene and the distance from the transcription start site.
