# Supplementary material for: The 5'-end transitional CpGs between the CpG islands and retroelements are hypomethylated in association with loss of heterozygosity in gastric cancers
Source: BMC Cancer. 2006 Jul 10;6:180. doi: 10.1186/1471-2407-6-180 (PMC1552088; doi:10.1186/1471-2407-6-180)
Supplement: Additional file 2 — Methylation alterations detected in 50 gastric cancers by methylation-specific PCR analysis and sequencing of common PCR DNA. CpG sites are indicated by the name of the gene and the distance from the transcription start site. One-level methylation alterations were analysed by the methylation-specific PCR intensity and the common PCR DNA. [file 1471-2407-6-180-S2.doc]

**Additional file 2 - Methylation alterations detected in 50 gastric cancers by methylation-specific PCR analysis and sequencing of common PCR** DNA

| CpG sites* | Methylation-specific PCR | | | |  | Common PCR DNA | | | |
| --- | --- | --- | --- | --- | --- | --- | --- | --- | --- |
|  | Total | No  alteration | One-level  alteration | ≥Two-level  alteration |  | Total | 21-40%  alteration | 41-60%  alteration | 0-20%  alteration |
| *CDH1*, 0 kb | 50 | 44 | 6 |  |  |  |  |  |  |
| *RABGEF1*, -0.2 kb | 50 | 47 | 3 |  |  |  |  |  |  |
| *STAG1*, -0.4 kb | 50 | 48 | 2 |  |  |  |  |  |  |
| *MYBPC2*, -1.2 kb† | 50 | 38 | 11 | 1 |  | 6 | 6 |  |  |
| -0.6 kb | 50 | 37 | 10 | 3 |  |  |  |  |  |
| *VDR*, -0.7 kb† | 50 | 25 | 21 | 4 |  | 8 | 8 |  |  |
| +0.1 kb | 50 | 50 |  |  |  |  |  |  |  |
| *ESR2*, -0.9 kb | 50 | 34 | 14 | 2 |  |  |  |  |  |
| *MLH1*, -1.0 kb† | 50 | 17 | 21 | 12 |  | 8 | 7 | 1 |  |
| -0.6 kb† | 50 | 31 | 13 | 6 |  | 4 | 4 |  |  |
| *FLJ43855*, -1.1 kb | 50 | 23 | 20 | 7 |  |  |  |  |  |
| *PTEN*, -1.4 kb | 50 | 37 | 13 |  |  |  |  |  |  |
| -0.9 kb | 50 | 43 | 7 |  |  |  |  |  |  |
| *CDKN2A*, -1.5 kb† | 50 | 22 | 22 | 6 |  | 8 | 7 |  | 1 |
| 0 kb† | 50 | 40 | 7 | 3 |  | 4 | 4 |  |  |
| +0.8 kb | 50 | 23 | 23 | 4 |  |  |  |  |  |
| *PAX5*, -0.1 kb | 50 | 31 | 17 | 2 |  |  |  |  |  |
| *RUNX2*, -3.8 kb | 50 | 34 | 16 |  |  |  |  |  |  |
| -3.0 kb† | 50 | 28 | 18 | 4 |  | 8 | 6 | 1 | 1 |
| -0.7 kb | 50 | 28 | 18 | 4 |  |  |  |  |  |
| +1.6 kb | 50 | 28 | 18 | 4 |  |  |  |  |  |
| *RUNX3*, -1.7 kb† | 50 | 15 | 20 | 15 |  | 8 | 6 | 2 |  |
| -0.5 kb | 50 | 22 | 20 | 8 |  |  |  |  |  |
| -0.1 kb | 50 | 35 | 15 |  |  |  |  |  |  |
| +1.0 kb | 50 | 27 | 20 | 3 |  |  |  |  |  |
| *KIAA1752*, +0.4 kb | 50 | 30 | 19 | 1 |  |  |  |  |  |
| *MUC8*, +2.0 kb | 50 | 28 | 19 | 3 |  |  |  |  |  |
| *MAGEA2*, 0 kb† | 50 | 35 | 2 | 13 |  | 2 | 2 |  |  |
| *DDX53*, 0 kb | 50 | 26 | 16 | 8 |  |  |  |  |  |
| *TFF2*, -0.2 kb† | 50 | 28 | 21 | 1 |  | 8 | 7 |  | 1 |
| *SERPINB5*, -0.3 kb† | 50 | 25 | 20 | 5 |  | 8 | 6 | 1 | 1 |
| *MSLN*, -0.8 kb† | 50 | 23 | 20 | 7 |  | 8 | 8 |  |  |
| Total | 1600 | 1002 (63%) | 472 (30%) | 126 (7%) |  | 80 | 71 (89%) | 5 (6%) | 4 (5%) |

*CpG sites are indicated by the name of the gene and the distance from the transcription start site.

†One-level methylation alterations were analysed by the methylation-specific PCR intensity and the common PCR DNA.
